# Case report of a huge lower lip cancer successfully treated with intra-arterial infusion chemotherapy

**DOI:** 10.1016/j.ijscr.2020.04.034

**Published:** 2020-05-11

**Authors:** Yen Ting Sheen, Yu Yuan Chen, Maw Chang Sheen

**Affiliations:** a Division of Plastic and Reconstructive Surgery, Kaohsiung Medical University Hospital, Faculty of Medicine, College of Medicine, Kaohsiung Medical University, Kaohsiung, Taiwan; b Department of Surgery, Kaohsiung Medical University Hospital, Faculty of Medicine, College of Medicine, Kaohsiung Medical University, Kaohsiung, Taiwan

**Keywords:** Squamous cell carcinoma, Surgery, Lip cancer, Methotrexate, Case report

## Abstract

**Introduction:**

We present a difficult case of huge squamous cell carcinoma (SCC) of lower lip that was successfully treated by intra-arterial infusion with methotrexate (MTX).

**Presentation of case:**

This 42-year-old female patient present with a fungating lower lip SCC of approximately 10 × 5 cm in size. MTX 25 mg was infused continuously to each side of external carotid artery every 24 h using two portable pumps. Totally, MTX 300 mg was given over 6 days. After treatment initiation, the tumor shrank dramatically and disappeared completely 2 months after the therapy. The patient was now recurrence-free at the recent follow-up 4 and half years after therapy.

**Discussion:**

Intra-arterial infusion chemotherapy has the advantage of delivering a high concentration of anticancer drug to the lesion to induce a rapid shrinkage of the tumor and the side effects are limited. Intra-arterial infusion with MTX achieves good tumor response to lower lip cancer with excellent anatomical and functional preservation.

**Conclusion:**

This therapy may be a treatment option in lower lip cancers with unresectable lesions, or in those patients who are unwilling to undergo resection.

## Highlights

• For lower lip cancer, early surgical excision is the treatment of choice.
• A difficult case of huge SCC of the lower lip that was successfully treated by intra-arterial infusion with methotrexate.
• Intra-arterial infusion chemotherapy has the advantage of delivering a high concentration of anticancer drug to the lesion.
• Intra-arterial infusion achieves good tumor response to lip cancer with excellent anatomical and functional preservation.

Early surgical excision is the treatment of choice for lower lip squamous cell carcinoma (SCC). We present a difficult case of huge SCC of the lower lip that was successfully treated by intra-arterial infusion with methotrexate. The lesion was extensive, which made complete resection difficult. This case report is reported in line with the SCARE criteria [1].

**Fig. 1:**
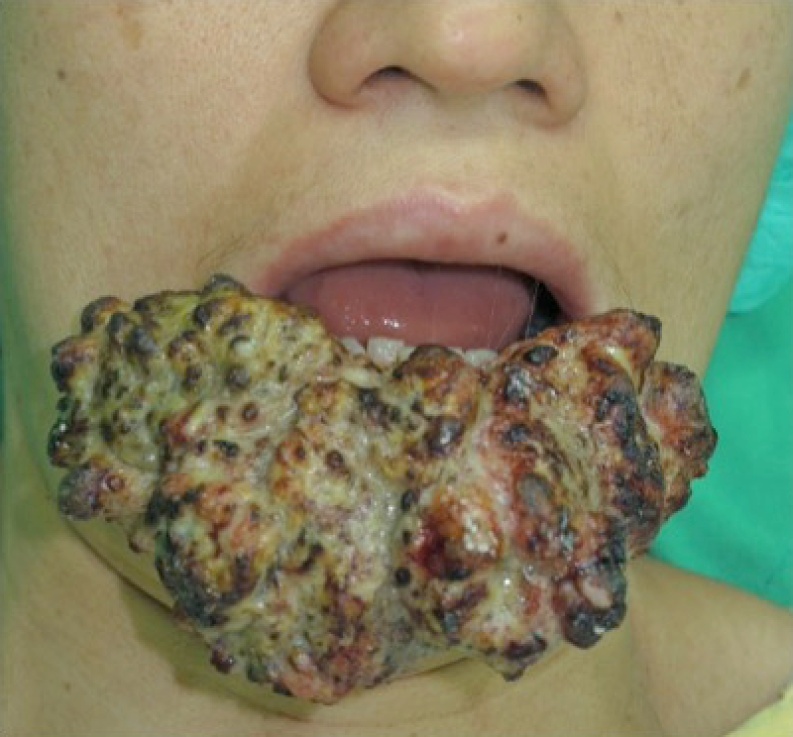
Lower lip SCC, before treatment (2015 May).

This 42-year-old female patient presented with a fungating lower lip mass of approximately 10 × 5 cm in size in May 2015. Two years before admission, a bean-sized tumor on her lower lip was noted and had been growing slowly for 2 years. Histopathologic examination showed grade 1 SCC. The patient declined surgical resection because of the extensive cosmetic and functional disability likely to result. She was admitted for intra-arterial infusion chemotherapy. There was no regional lymphadenopathy on admission. Systemic image survey including computed tomography (CT) and positron emission tomography (PET) showed no evidence of distant metastasis (Fig. 1).

**Fig. 2:**
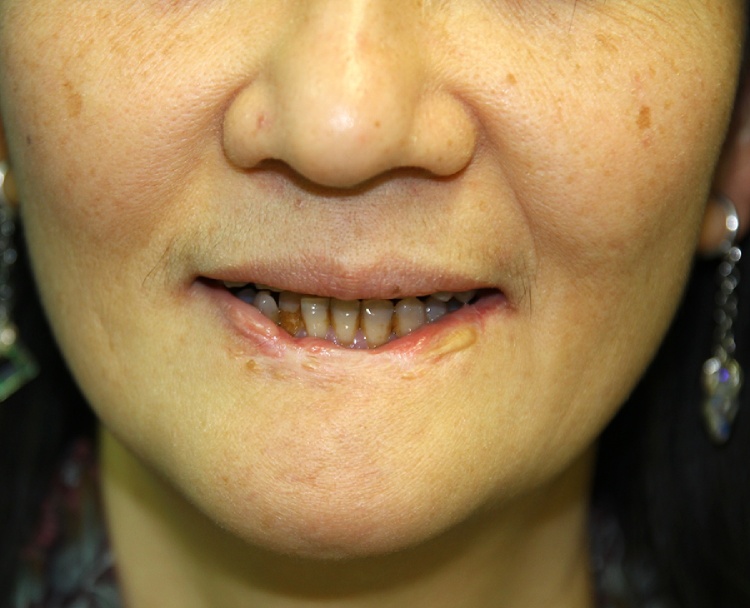
Lower lip SCC, four and half years after treatment (2020 January).

The implantable port-catheter system (Jet Port Plus Allround; PFM, Cologne, Germany) was used for catheterization. Under general anesthesia, the catheter was inserted through the right superficial temporal artery into the external carotid artery with the tip located proximal to the branching of the facial artery. The distal end was embedded subcutaneously along the lateral neck and connected to the port, which was implanted subcutaneously near the infra-clavicular region [2,3]. The same procedure was also performed on the left side. Methotrexate (MTX) 25 mg was infused continuously to each side of the external carotid artery every 24 h using two portable pumps (CADD-1; Deltec, St Paul, MN, U.S.A.). Citrovorum factor (15 mg) was given orally every 12 h during the period of MTX infusion. Totally, MTX300 mg was given over 6 days and stopped because of leukopenia (2920/μL, nadir 2200//μL) and elevation of GOT (534IU/L) and GPT (351IU/L). These side effects were grade 2 according to WHO classification. No other adverse event was noted. After treatment initiation, the tumor shrank dramatically and disappeared completely 2 months after therapy. No further anticancer therapy was given. The patient was followed at the outpatient clinic regularly. The patient was now recurrence-free at the recent follow-up 4 and half years after therapy (January 2020) (Fig. 2).

Intra-arterial infusion chemotherapy has the advantage of delivering a high concentration of anticancer drug to the lesion to induce a rapid shrinkage of the tumor. Intra-arterial infusion with MTX achieves good tumor response to early lower lip cancer with excellent anatomical and functional preservation [4]. The unique results of this case and our previously reported case of lower lip verrucous carcinoma [5] show this therapy is effective to both early and advanced cancers of the lower lip. This may be a treatment option in lower lip cancers with unresectable lesions, or in those patients who are unwilling to undergo resection.

Written informed consent was obtained from the patient for publication of this case report and accompanying images. A copy of the written consent is available for review by the Editor-in-Chief of this journal on request.

## Declaration of Competing Interest

No conflicts of interest.

## Funding

No funding of this research.

## Ethical approval

This study is exempt from ethical approval.

## Consent

Fully informed patient consent was obtained and provided.

## Author contribution

Study concept and design of study: M.C. Sheen.

Data collection: Y.T. Sheen, Y.Y. Chen.

Data analysis and interpretation: Y. T. Sheen, M.C. Sheen.

Writing the paper: Y.T. Sheen.

Revising the manuscript critically for important intellectual content: M.C. Sheen, Y.T. Sheen.

## Registration of research studies

This study don’t involve the human participants.

## Guarantor

Y.T. Sheen.

Y.Y. Chen.

M.C. Sheen.

## Provenance and peer review

Not commissioned, externally peer-reviewed.
